# The Effect of Emojis on L2 Irony Processing for Chinese EFL Learners

**DOI:** 10.1002/pchj.70116

**Published:** 2026-08-10

**Authors:** Wan Su, Hadina Habil

**Affiliations:** ^1^ Language Academy Universiti Teknologi Malaysia Skudai Malaysia

**Keywords:** Chinese EFL learners, embodied cognition, emojis, L2 irony

## Abstract

Second language (L2) learners often struggle to draw on embodied emotional experiences in the target language, which impedes their rapid and accurate interpretation of nonliteral ironic meaning. Grounded in embodied cognition theory, the present study examined how emojis and the valence congruity between emojis and contextual information modulate Chinese EFL learners’ comprehension of positive and negative irony in computer‐mediated communication. The results of this study revealed that emojis improved accuracy in negative—but not positive—irony comprehension, whereas valence congruity enhanced accuracy in positive—but not negative—irony. Moreover, both the presence of emojis and valence congruity accelerated irony processing. These findings indicate that emojis and their emotional valences facilitate L2 irony processing by bolstering learners’ sensitivity to speakers’ affective states, and offer important implications for L2 discourse processing research from an embodied cognition perspective.

## 1 Introduction

Irony is a common form of figurative language, characterized by a contradiction between the literal meaning and the speaker's communicative intention. This indirect mode of communication often conveys humor, sarcasm, or criticism (Attardo 2000). Irony can be divided into positive irony and negative irony. The former involves saying something negative to express a positive evaluation or praise, making the intended meaning more concealed and harder for the listener to recognize. Such expressions are easily misinterpreted as impolite, and their comprehension requires shared knowledge and familiarity with the preceding context (situation). Negative irony, on the other hand, appears to be praise but actually conveys criticism, reflecting the speaker's negative emotions or disapproval (Harris and Pexman 2003). Compared to positive irony, negative irony is more conventional and frequently used, allowing listeners to more easily detect the semantic reversal between literal meaning and intended message by integrating linguistic and non‐linguistic cues (Ellis et al. 2021; Pexman and Olineck 2002).

Irony is particularly demanding for L2 learners because they must move beyond the literal form of an utterance and integrate contextual, pragmatic, and socio‐cultural information. Existing accounts emphasize different parts of this process. From a pragmatic perspective, irony requires readers to detect a discrepancy between what is said and what is intended, increasing inferential load (Grice 1975). From a socio‐cultural perspective, learners' success also depends on how familiar they are with the situations, norms, and cueing practices that make irony appropriate in a given community (Ellis et al. 2021; Shively et al. 2022). These accounts jointly suggest that L2 irony comprehension is not only a matter of linguistic decoding but also a process of integrating literal meaning, contextual expectations, and social‐pragmatic cues.

In computer‐mediated written communication, many facial, prosodic, and bodily cues are absent. Emojis may therefore serve as paralinguistic cues that help readers infer the sender's emotional stance and communicative intention (Boutet et al. 2021; Garcia et al. 2022). However, their effect is unlikely to be uniform. Hand et al. (2022) showed that the interpretation of messages depends on the relation between text content and emoji type, suggesting that emoji‐context congruence is a key variable. In the present study, emoji presence refers to whether an emotional emoji accompanies the target utterance, whereas emoji‐context congruence refers to the degree to which the emoji's emotional valence is consistent with the emotional information implied by the situation. This distinction allows us to test whether emojis merely provide an additional cue or whether their fit with the context changes L2 irony processing. Recent cross‐cultural evidence further indicates that the facilitative effect of emojis on irony comprehension is culturally patterned: smiling emojis tend to aid irony comprehension in Eastern contexts, whereas winking emojis are more effective in Western contexts (Du 2025; Du et al. 2026). These findings suggest that emoji interpretation is not universal but is shaped by culturally grounded pragmatic conventions, underscoring the need to examine how Chinese EFL learners specifically deploy emoji cues during L2 irony resolution.

Embodied cognition provides the theoretical basis for predicting why emojis may affect L2 irony processing. According to this view, language comprehension is grounded in sensorimotor and affective experience (Ellis 2019; Schneegans and Schöner 2008). Perceptual‐symbol accounts further propose that linguistic meaning is supported by the partial reactivation of prior perceptual and emotional states (Barsalou 1999). Evidence from metaphor and humor processing shows that bodily states such as facial expressions and movements can influence language interpretation (Wilson and Gibbs 2007; Xu et al. 2023). Because irony often involves an affective contrast between literal wording and intended meaning, emojis may provide visible emotional cues that help L2 learners construct or re‐enact the speaker's affective stance (Filik et al. 2015).

Taken together, pragmatic, socio‐cultural, and embodied accounts converge on the prediction that L2 irony comprehension depends on the integration of literal wording, contextual information, and emotional cues. The present study therefore examined Chinese university EFL learners in CMC contexts and asked two questions: (1) Does the presence of an emoji influence the accuracy and speed of L2 irony processing? (2) Does the congruence between emoji valence and contextual information modulate the accuracy and speed of L2 irony processing? Based on the literature reviewed above, we expected emojis to facilitate irony processing, especially when their emotional valence was congruent with the contextual information.

## 2 Experiment 1

A 2 × 4 within‐subjects design was used in Experiment 1 to test how emoji availability influences L2 irony understanding. Participants saw sentences that either included or excluded an emoji, and these sentences fell into four categories: positive irony, negative irony, positive literal meaning, and negative literal meaning. We measured both the accuracy and the speed of their interpretive judgments.

### 2.1 Method

#### 2.1.1 Participants

An a priori power analysis using G*Power 3.1.9.7 (Faul et al. 2009) showed that a repeated‐measures ANOVA with two (emoji: present vs. absent) × four (sentence type) within‐subject cells requires at least 22 participants to detect a medium effect of *f* = 0.25 at *α* = 0.05 and 95% power. Twenty‐eight university students (13 female and 15 male, age: 17–22 years, *M* = 18.79, SD = 1.10) took part in Experiment 1. None majored in English; their English scores on the National College Entrance Examination ranged from 110 to 135, indicating that they could read the literal content of the materials. All participants were native Mandarin speakers who had learned English primarily through formal classroom instruction. They reported limited opportunities for immersive English communication outside the classroom. Participants' familiarity with the specific emojis used in the experiment was not formally assessed, nor were their pragmatic competence or prior experience with verbal irony measured. These variables may influence irony comprehension (Wang et al. 2023) and should be considered when interpreting the results. The sample size (*N* = 28) was determined by an a priori power analysis, but it nevertheless limits the generalisability of the findings to broader populations of Chinese EFL learners. Participants reported normal or corrected‐to‐normal vision and no history of language or neurological disorders. Everyone was in good health and provided written consent prior to the experiment.

#### 2.1.2 Materials

Following Ellis et al. (2021), we constructed 64 items, each consisting of a scenario, a target utterance, and a verification sentence. The items were evenly distributed across four sentence types: positive irony, negative irony, positive literal meaning, and negative literal meaning. Within each sentence type, half of the items contained an emoji and half did not (see Table 1). Emoji valence was consistent with the overall contextual valence in the emoji‐present condition (positive irony emoji 😀 for positive irony, negative irony emoji 😠 for negative irony). Item length did not differ significantly across categories (*p* > 0.05). The materials were adapted from Ellis et al. (2021) and pilot‐tested with a separate group of 10 Chinese EFL learners (not included in the main experiment) to ensure clarity and cultural appropriateness. These pilot participants rated the comprehensibility of each scenario on a 5‐point scale (1 = *very difficult to understand*, 5 = *very easy*), and items with mean ratings below 3 were revised. The final set of materials was not subjected to a formal norming study for irony detection, which is a limitation acknowledged in the General Discussion.

**TABLE 1:** A material example in Experiment 1.

Situation	Target utterance	Type
Heather is late meeting up with Sam. When she finally arrives Sam says	Right on time again Heather. 😠	Negative irony
Dave has just bought a new sports car and is taking his friend Sam for a drive. Dave drives crazily fast and Sam is impressed. Sam says to Dave	This car is a bit on the slow side, isn't it? 😀	Positive irony
Peter has been trying to date Jenefer. Jenefer has politely refused all his invitations but Peter carries on asking for a date. Jenefer says	You never give up, do you? 😠	Negative literal
Maggie is looking very smart in her new formal suit. When her friend Simon sees her he says	You're a fashion queen, Maggie.😀	Positive literal

#### 2.1.3 Procedures

**FIGURE 1:**
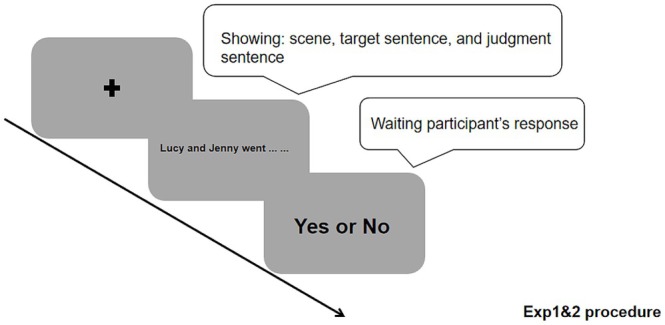
The procedure of an example trial of Experiment 1 and 2.

The experiment (see Figure 1) was conducted individually in a quiet room. The session comprised a practice block and a main experimental block programmed in PsychoPy 2025.2.0 (Peirce 2007), presented on a 17‐in. CRT monitor (resolution: 1024 × 768, refresh rate: 85 Hz). Participants were seated approximately 60 cm from the screen with their eyes aligned to the center of the display.

Before the main task, participants completed a practice block of 8 trials to familiarize themselves with the self‐paced semantic judgment task. The practice trials covered all four sentence types (positive irony, negative irony, positive literal, and negative literal), including emoji‐present conditions. Each trial began with a black fixation cross “+” (Song font, 24 pt., visual angle ≈ 0.8°, white background) displayed at the center of the screen for 500 ms. This was followed by the sequential presentation of the scenario, target sentence, and verification sentence (all in black Song font, 24 pt., visual angle ≈ 1.5°, 1.5 line spacing, white background). The order of trials was fully randomized, with a pseudo‐random constraint preventing more than three consecutive trials from the same condition. Participants read the materials at their own pace and classified the verification sentence as correct (“Y”) or incorrect (“N”) using a standard QWERTY keyboard. They were instructed to respond using the index finger of their dominant hand throughout the experiment. All text remained on the screen until a response was registered; if no response was made within 10 s, the trial automatically advanced to the next. The software recorded both accuracy (scored as 1 for correct and 0 for incorrect) and reaction times (RTs), measured from the onset of the verification sentence until the participant's keypress (recorded in milliseconds and subsequently converted to seconds). The main task comprised 64 trials in Experiment 1. A 2–3 min break was provided after half of the trials were completed. The entire session lasted approximately 30 min. For the accuracy analysis, correct responses received 1 point and incorrect responses received 0 points, yielding a maximum score of 8 per condition. Reaction times were computed from the onset of the verification sentence until the judgment key was pressed.

#### 2.1.4 Transparency and Openness Statement

The complete dataset is available via the Open Science Framework (https://osf.io/za2qd). This study's design and hypotheses were not preregistered.

### 2.2 Results

Practice data were excluded from subsequent analyses. Following Ellis et al. (2021), three participants whose overall accuracy was below 50% were excluded from all analyses. Accuracy analyses were conducted on the remaining valid trials. For reaction‐time analyses, trials with incorrect responses (10.76%) and outlying latencies (< 300 ms or > 3 SD from each participant's mean; 1.02%) were excluded (Wang et al. 2023).

**FIGURE 2:**
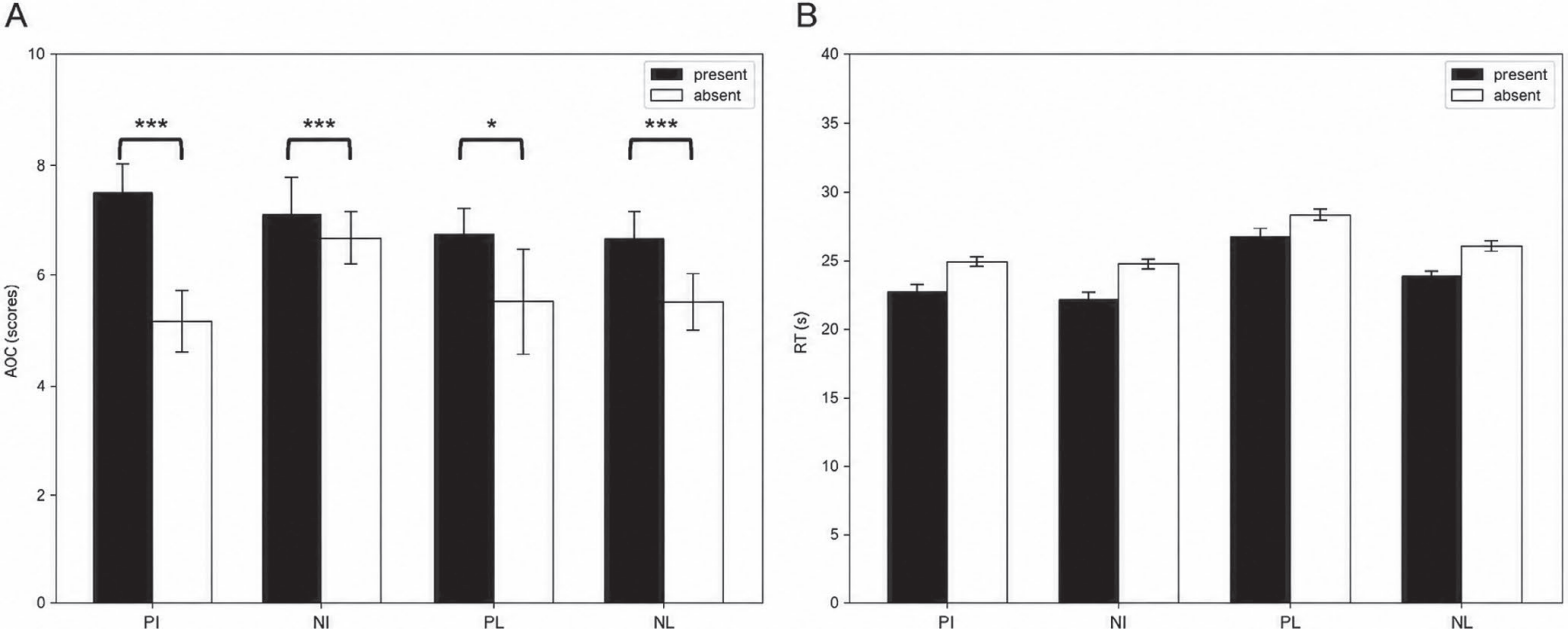
The effect of emoji presence on sentence processing accuracy and reaction time across four sentence types. “PI” is positive irony, “NI” is negative irony, “PL” is positive literal, “NL” is negative literal; “present” represents “with emoji”, “absent” represents “without emoji”. Note: N.s represents *p* > 0.05, * represents *p* < 0.05, and *** represents *p* < 0.001.

To examine how the presence or absence of emojis affects accuracy and response time across the four sentence types, we conducted a 2 (emoji: present vs. absent) × 4 (sentence type: positive irony, negative irony, positive literal, negative literal) repeated‐measures ANOVA using the “manova“ and “emmeans” functions from the BruceR package in R (Shuang 2023). Because the semantic‐judgment response times were not normally distributed, a log transformation was applied before further analysis. The outcomes are displayed in Figure 2.

For accuracy (Figure 2A), the 2 × 4 repeated‐measures ANOVA showed significant main effects of emoji (*F*(1, 24) = 45.07, *p* < 0.001, *partial η* ^*2*^ = 0.65) and sentence type (*F*(3, 72) = 32.20, *p* < 0.001, *partial η* ^*2*^ = 0.57), as well as a significant emoji × sentence type interaction (*F*(3, 72) = 30.31, *p* < 0.001, *partial η* ^*2*^ = 0.56). Bonferroni‐corrected follow‐up comparisons showed that emoji presence was associated with higher accuracy for negative irony (*p* < 0.001), positive literal sentences (*p* = 0.026), positive irony (*p* < 0.001), and negative literal sentences (*p* < 0.001). Because the interaction indicates that the size of the emoji effect varied across sentence types, we focus on the theoretically central negative‐irony contrast: accuracy was higher with emojis than without emojis (*t*(24) = 5.25, *p* < 0.001, *Cohen's d* = 1.04).

For response times (Figure 2B), the ANOVA showed significant main effects of emoji (*F*(1, 24) = 31.69, *p* < 0.001, *partial η* ^*2*^ = 0.57) and sentence type (*F*(3, 72) = 10.56, *p* < 0.001, *partial η* ^*2*^ = 0.31), whereas the interaction was not significant (*p* > 0.05). Bonferroni‐corrected comparisons indicated that responses were faster when an emoji was present than when it was absent (*t*(24) = −3.79, *p* < 0.001, *Cohen's d* = 0.76).

## 3 Experiment 2

In Experiment 2, a 2 × 3 within‐participants design was used to further investigate the impact of emoji‐context valence alignment on L2 irony understanding. Participants encountered positive or negative ironic sentences followed by emojis that matched, partially matched, or mismatched the prevailing emotional context; their interpretive accuracy and speed were recorded.

### 3.1 Method

#### 3.1.1 Participants

Twenty‐eight university students (14 female and 14 male, age: 17–22 years, *M* = 19.25, SD = 1.38) took part in Experiment 2, and none had taken part in Experiment 1. All were non‐English majors whose English scores on the National College Entrance Examination ranged from 110 to 135, confirming effortless comprehension of the literal content of the materials. As in Experiment 1, participants were native Mandarin speakers with classroom‐based English learning backgrounds. Their familiarity with the six emojis used in Experiment 2 was not assessed prior to the main task, although a separate group of 20 students rated the emotional valence of these emojis. Individual differences in emoji usage frequency and pragmatic competence were not measured, which may moderate the observed effects and is acknowledged as a limitation. They reported good health and signed an informed‐consent form before testing began.

#### 3.1.2 Materials

Materials for Experiment 2 were again modeled on Ellis et al. (2021). Each item comprised a scenario, a target sentence and a test sentence; an emoji always appeared at the end of the target sentence. Forty‐eight items were constructed (see Table 2). The congruence between the emoji's emotional valence and the contextual information was manipulated at three levels—congruent, weakly congruent and incongruent—with equal numbers of positive‐ and negative‐irony sentences in each level. This yielded six item types: positive irony with congruent/weakly congruent/incongruent emoji, and negative irony with congruent/weakly congruent/incongruent emoji. All items were presented in random order and the six subsets did not differ in length (*p* > 0.05).

**TABLE 2:** A material example in Experiment 2.

	Positive irony	Negative irony
Situation	Leon has just purchased a new computer. He invites his classmate David to test the new machine. David conducts many high‐intensity tasks by this computer and it runs smoothly, Leon says to David	Tony promised that he would prepare an excellent birthday gift for his friend Lisa this year. When the date was arriving, Tony focused on his own work and forgot it. Lisa said
Congruent target utterance	The computer responds slowly, doesn't it? 😀	What a wonderful birthday experience! 😠
Weakly congruent target utterance	The computer responds slowly, doesn't it? 😉	What a wonderful birthday experience! 😐
Incongruent target utterance	The computer responds slowly, doesn't it? 😒	What a wonderful birthday experience! 🥺

#### 3.1.3 Procedures

The emotional valence of emojis was determined based on ratings from 20 college students on six emojis using a 5‐point scale (1 = *very positive*, 2 = *slightly positive*, 3 = *neutral*, 4 = *slightly negative*, 5 = *very negative*). Over 80% of the students rated the “broad smile” emoji (😀) as 1, the “angry” emoji (😠) as 5, the “winking smile” emoji (😉) as 2, and the “pouting” emoji (😐) as 4. Additionally, approximately 50% of the students rated the “frowning cute” emoji (🥺) as 2 and the “sneering” emoji (😒) as 4. Therefore, we ultimately selected 😀, 😉, and 😒 as the emojis for the congruent, weakly congruent, and incongruent conditions of positive irony, respectively, and 😠, 😐, and 🥺 as the emojis for the congruent, weakly congruent, and incongruent conditions of negative irony (see Table 2). None of the students who participated in the emoji valence rating took part in the semantic judgment task. The rating task provided a manipulation check for emoji valence before the main experiment. However, because familiarity with specific emojis and individual pragmatic ability were not measured in the main participant group, these variables were not included as covariates; this limitation is acknowledged in “General Discussion”. The main task comprised 48 trials in Experiment 2. The procedures of Experiment 2 were identical to those of Experiment 1, with the exception that all target utterances included an emoji and the verification task remained a binary semantic judgment. The self‐paced reading paradigm with binary judgments, while widely used in L2 irony research (Ellis et al. 2021), does not capture the moment‐by‐moment integration of cues; this methodological constraint is discussed in the General Discussion.

### 3.2 Results

Experiment 2 followed the same analytic procedure as Experiment 1. Every participant scored above 50% on the semantic judgements and was retained. Accuracy analyses included all valid trials from retained participants. For reaction‐time analyses, trials with incorrect responses (8.75%) and outlying latencies (> 3 SD from each participant's mean or < 300 ms; 0.53%) were excluded.

**FIGURE 3:**
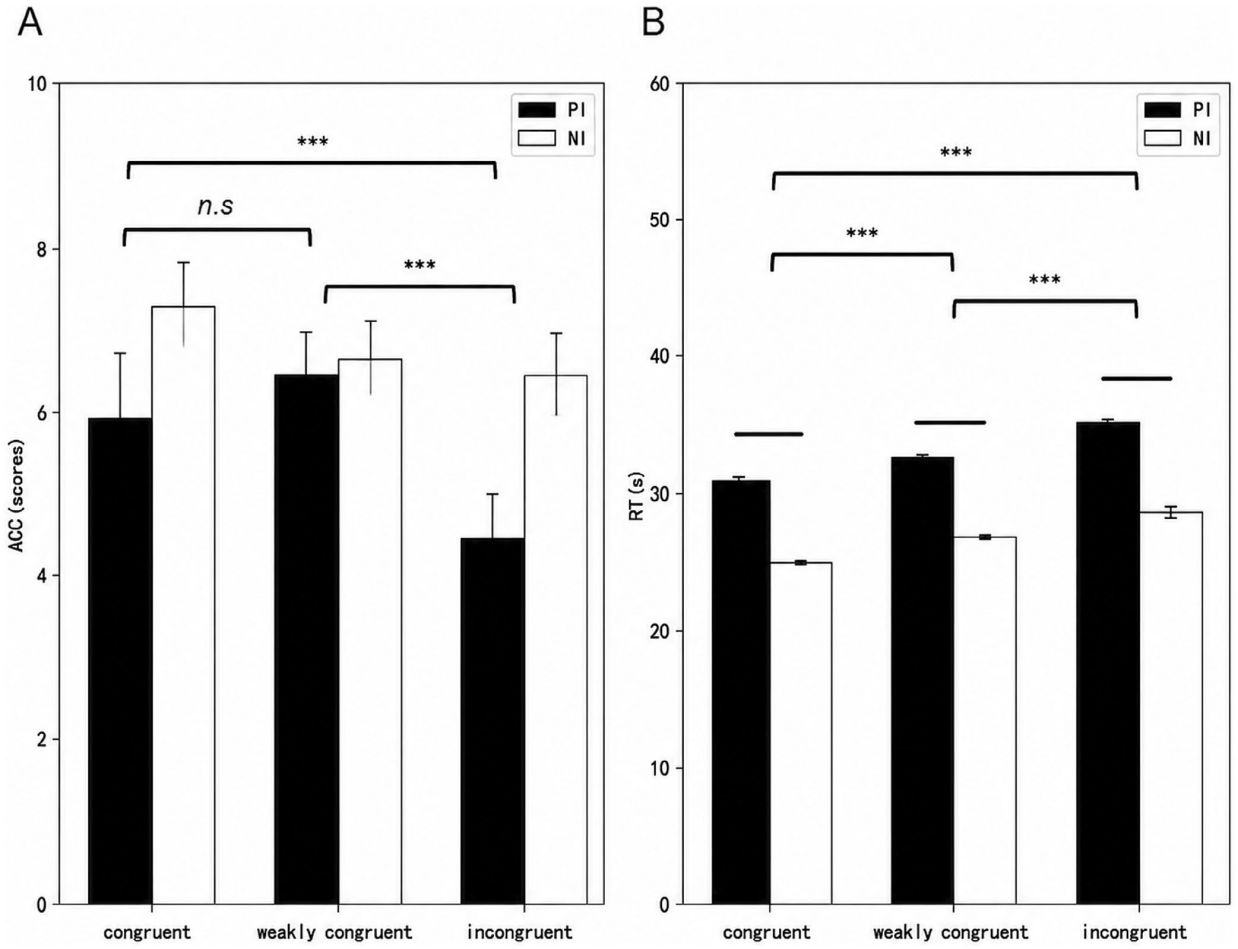
The effect of the degree of coordination between the valence of emojis and contextual information on the accuracy of irony processing and reaction time. “PI” is positive irony, “NI” is negative irony. Note: N.s represents *p* > 0.05, *** represents *p* < 0.001.

To examine how the congruence between emoji emotional valence and contextual information affects learners' irony comprehension accuracy and response times, we conducted a 2 (irony type: positive vs. negative) × 3 (congruence: congruent vs. weakly congruent vs. incongruent) repeated‐measures ANOVA using the “manova” and “emmeans” functions from the BruceR package in R. The results are presented in Figure 3, which illustrates the effects of different degrees of emoji‐context congruence on irony processing accuracy and response times.

For accuracy (Figure 3A), the 2 × 3 repeated‐measures ANOVA showed significant main effects of congruence (*F*(2, 54) = 12.49, *p* < 0.001, *partial η* ^*2*^ = 0.32) and irony type (*F*(1, 27) = 10.45, *p* < 0.001, *partial η* ^*2*^ = 0.28), as well as a significant interaction (*F*(2, 54) = 19.40, *p* < 0.001, *partial η* ^*2*^ = 0.46). Follow‐up comparisons indicated that congruence was associated with accuracy for positive irony (*p* < 0.001), but not for negative irony (*p* > 0.05). Bonferroni‐adjusted comparisons for positive irony showed higher accuracy in the congruent than incongruent condition (*t*(27) = 5.18, *p* < 0.001, *Cohen's d* = 0.98) and in the weakly congruent than incongruent condition (*t*(27) = 3.59, *p* < 0.001, *Cohen's d* = 0.68), whereas the congruent and weakly congruent conditions did not differ (*p* > 0.05).

For response times (Figure 3B), the ANOVA showed significant main effects of irony type (*F*(1, 27) = 35.76, *p* < 0.001, *partial η* ^*2*^ = 0.57) and congruence (*F*(2, 54) = 51.20, *p* < 0.001, *partial η* ^*2*^ = 0.69), whereas the interaction was not significant (*p* > 0.05). Bonferroni‐adjusted comparisons indicated faster responses in the congruent than weakly congruent condition (*t*(27) = −4.20, *p* < 0.001, *Cohen's d* = 0.79), faster responses in the congruent than incongruent condition (*t*(27) = −2.54, *p* < 0.001, *Cohen's d* = 0.48), and faster responses in the weakly congruent than incongruent condition (*t*(27) = −5.64, *p* < 0.001, *Cohen's d* = 1.07). Thus, the degree of valence alignment between emoji and context reliably modulated L2 irony processing speed.

## 4 General Discussion

This study examined whether emoji presence (Experiment 1) and emoji‐context congruence (Experiment 2) were associated with Chinese EFL learners' accuracy and response speed in irony processing during CMC. Experiment 1 showed that emoji presence improved accuracy for negative irony and accelerated semantic judgments overall. Experiment 2 showed that stronger emoji‐context congruence improved accuracy for positive irony and was associated with faster irony processing. The discussion below interprets these patterns cautiously in relation to the statistical outcomes and the theoretical accounts reviewed above.

From an embodied‐cognition perspective, understanding communicative intent benefits from the joint contribution of situation, linguistic context, and paralinguistic cues. Experiment 1, however, showed that emojis only enhanced accuracy for negative irony; positive‐irony accuracy remained unchanged. This asymmetry probably reflects the greater difficulty of positive irony (Ellis et al. 2021; Pexman and Olineck 2002). First, positive irony violates more conversational maxims and therefore contradicts the addressee's expectations more strongly. Negative irony (“saying something positive while meaning something negative”) infringes only Grice's Quality Maxim, whereas positive irony (“saying something negative while meaning something positive”) simultaneously violates Quality and the Politeness Principle (Grice 1975). The more maxims are breached, the harder it is for learners to infer the implied meaning. Second, positive irony is less conventional and less frequent than negative irony, so L2 learners encounter it less often and find it harder to detect the semantic reversal between the target sentence and the preceding context.

As positive and negative irony differ in processing difficulty, non‐verbal cues such as emojis make distinct contributions to each. For the less demanding negative irony, emojis improved accuracy. This benefit may be accounted for within the embodied‐cognition‐inspired theory of perceptual symbol systems, which holds that sensory information (visual, auditory, tactile, etc.) constitutes a core resource for comprehension (Barsalou 1999). Perceptual symbols are re‐enactive representations stored in long‐term memory; when relevant cues are encountered, these simulations may be re‐activated and facilitate linguistic processing. For negative irony, an emoji conveying negative affect may have provided an additional emotional cue that was consistent with the intended critical meaning, thereby potentially supporting learners' interpretation. However, because the present study did not directly measure embodied simulation (e.g., through facial EMG or body movement tracking), this interpretation remains speculative and awaits empirical verification through process‐sensitive methodologies in the future. For positive irony, the positive emoji alone may not have been sufficient for learners to override the negative literal form and recover the intended praise. Thus, the present data suggest that emojis can support interpretation when the intended meaning is already relatively accessible, but this conclusion should be tested with process‐sensitive measures in future research.

Participants were faster to judge the meaning of ironic sentences when an emoji was present than when it was absent, a pattern that aligns with previous work on discourse processing (Boutet et al. 2021; Garcia et al. 2022). The RT results in this current study are consistent with the possibility that emojis reduced some of the inferential effort required during semantic judgment (Boutet et al. 2021; Garcia et al. 2022). Ellis et al. (2021) proposed that learners first compute a literal interpretation, then detect a mismatch between literal meaning and context, and finally infer the speaker's intended meaning. Within this account, emojis may help constrain the final inferential step by making the speaker's emotional stance more visible. Because the present study used self‐paced reading and binary judgments, however, it cannot determine the precise time course of this process. Future studies using eye‐tracking or EEG are needed to test this claim directly.

The results of Experiment 2 show that the semantic fit between emoji valence and context improved accuracy for positive irony, yet left negative‐irony accuracy unchanged. Again, the likely reason is that positive irony is inherently harder to process (Ellis et al. 2021; Pexman and Olineck 2002). Crucially, Experiment 2 introduced three degrees of fit—congruent, weakly congruent, and incongruent—creating a more complex cue landscape. For the relatively easy negative‐irony items, learners could still infer the intended criticism by detecting the reversal between context and target sentence. Although the emoji activated an embodied affective trace, participants noticed that its valence was not always reliable; when it conflicted with the context, they discounted it and based their interpretation on the linguistic cues alone. Consequently, varying degrees of emoji‐context congruence did not modulate accuracy for negative irony.

In contrast, the more demanding positive‐irony items prevented learners from identifying the reversal on the basis of context alone. Even when the emoji valence was not fully aligned with the context, this mismatch was difficult to detect. Under these conditions, learners relied on the salient emotional signal provided by the emoji to infer the speaker's meaning, resulting in higher accuracy when the emoji and context were congruent. This pattern aligns with the Graded Salience Hypothesis: more salient information is recruited first to compute meaning (Giora 1999). When contextual evidence is clear, it is used preferentially; when it is insufficient, salient non‐verbal cues such as emojis are recruited instead. Thus, the data illustrate the dynamic and competitive nature of language processing, in which linguistic and paralinguistic sources continuously interact and sometimes compete for dominance. Experiment 2 further showed that the better the emoji's emotional valence matched the context, the faster learners processed irony. Again, this can be interpreted within the L2 irony roadmap and the embodied‐cognition‐inspired theory of perceptual symbols (Barsalou 1999; Ellis et al. 2021). As noted above, the final step—inferring the speaker's intended meaning—imposes the heaviest cognitive load. We speculate that an emoji may rekindle the relevant affective simulation; when its valence is congruent with that simulation, the inferential cost may be reduced and the response may become quicker. Direct evidence for this load‐reduction account awaits eye‐tracking or EEG studies, as the present design cannot isolate the contribution of embodied simulation from other cognitive processes. Overall, emojis and contextual information jointly shape L2 irony comprehension. First, when learners can already detect the semantic reversal between the target sentence and the preceding scenario, emojis boost accuracy; when the reversal is hard to spot, emojis provide no such gain. Second, under conditions of complex emoji–context valence mapping, learners default to the linguistic context and turn to emojis only when contextual cues prove insufficient. Third, as long as emoji valence is at least partly aligned with the context, processing speed increases. Thus, L2 irony resolution is a dynamic trade‐off in which the relative weight of emoji versus context is continuously recalibrated according to the clarity of the linguistic information and the degree of emoji–context congruence.

Beyond these general mechanisms underlying L2 irony processing, the current findings also reveal distinct patterns specific to Chinese EFL learners—patterns shaped by their unique learning contexts, cultural values, and pragmatic habits. This specificity not only enriches the empirical implications of our study but also addresses the core concern of contextualizing L2 research to non‐Western learner groups.

Chinese EFL learners predominantly acquire English through classroom‐based instruction, with limited opportunities for immersive embodied emotional experiences in the target language (Guan 2019). Unlike learners in English‐speaking countries who are exposed to oral irony with natural paralinguistic cues (e.g., intonation, facial expressions) in daily interactions, Chinese learners rely heavily on computer‐mediated communication (CMC) (e.g., online English courses, cross‐border academic discussions, social media) as a key channel to engage with English discourse. This context suggests that emojis may serve as a compensatory non‐verbal cue—they may fill the gap left by the absence of real‐time emotional signals in written CMC, potentially activating learners' embodied emotional simulations more effectively than in other learning contexts. It is important to note that this interpretation is speculative because the present study did not directly compare Chinese learners with other learner groups or measure their CMC usage patterns. Consistent with this, the present study found that emojis significantly improved the accuracy of negative irony comprehension and accelerated overall processing speed for Chinese learners. This pattern is compatible with previous evidence that emojis can support sarcasm comprehension in some reader groups (Garcia et al. 2022), but direct cross‐study comparisons should be made cautiously because participant populations and task designs differ. For Chinese EFL learners, even in normal cognitive states, emojis serve as “emotional anchors” that strengthen their perception of the speaker's intent—especially for negative irony, which is more familiar in Chinese daily communication (Wang et al. 2023)—confirming that emojis play a more indispensable role in supporting L2 irony processing for Chinese learners due to their limited embodied exposure to English.

The findings may also reflect the pragmatic experience of Chinese EFL learners. Wang et al. (2023) suggest that Chinese learners' processing of L2 irony is shaped by proficiency and pragmatic experience, and Du et al. (2024) further show that cultural factors can affect irony comprehension. On this basis, Chinese EFL learners may be more familiar with negative irony than positive irony, which could help explain why emoji presence facilitated negative‐irony accuracy in Experiment 1, whereas emoji‐context congruence was needed to support positive‐irony accuracy in Experiment 2. This interpretation remains highly speculative because the present study did not directly measure participants' prior experience with irony, emoji familiarity, or cultural‐value orientation.

Compared with similar international studies, the results for Chinese EFL learners show distinct cross‐cultural differences. Krygier‐Bartz and Glenwright (2022) conducted a comparative study on English as an Additional Language (EAL) learners including Korean backgrounds and Chinese EFL learners, finding that Korean EAL learners' accuracy in understanding English negative irony was 15% lower than that of Chinese learners. This gap stems from the fact that the frequency of irony (sarcasm) in Korean culture is less than 1/5 of that in English culture, while Chinese culture's frequent use of negative irony provides pragmatic transfer advantages for Chinese EFL learners. In contrast to American native speakers, Chinese learners' overall irony comprehension level is lower (Du et al. 2024), but the facilitative effect of emojis is more obvious—this is because American individualistic culture emphasizes direct expression, and irony is often used for humor or criticism with clear cues, while Chinese collectivist culture relies more on external emotional cues (such as emojis) to disambiguate indirect expressions. These differences suggest that the role of emojis in L2 irony processing may not be universal but may be modulated by learners' cultural background. However, these cross‐cultural comparisons are post hoc interpretations based on existing literature rather than direct comparisons in the present study, and should be treated as generative hypotheses for future research. These findings have important theoretical implications: the facilitative effect of emojis on L2 irony processing is shaped by the interaction of learning context and cultural values. For Chinese EFL learners, emojis act as a “bridge” between linguistic form and embodied emotion, compensating for their lack of target language immersion and resolving ambiguity aversion caused by uncertainty avoidance, which enriches the embodied cognition theory in L2 research by highlighting the cultural specificity of non‐verbal cue processing.

Recent studies employing Chinese–English bilingual or cross‐cultural designs further corroborate the culturally modulated role of emojis in irony processing (Du 2025; Du et al. 2026). Specifically, Du (2025) demonstrated that Chinese participants showed preferential reliance on smiling emojis for irony comprehension, whereas American participants benefited more from winking emojis. Extending this line, Du et al. (2026) found that culturally preferred emojis not only enhanced real‐time irony comprehension but also improved later recognition memory, supporting a multimodal account in which emojis contribute verbal, social, and visual cues to the processing of non‐literal intent. These findings align with the present observation that emoji facilitation is not uniform: Chinese EFL learners in our study showed heightened sensitivity to negative emojis for negative irony and relied on emoji‐context congruence to resolve positive irony, suggesting that L2 learners' emoji use is constrained by both their L1 pragmatic experience and the specific emotional valence signaled by the emoji.

The specificity of the results also provides targeted insights for Chinese EFL teaching. Given that CMC is a major context for Chinese learners to use English, teachers can incorporate emoji‐based training into pragmatic instruction. For negative irony, educators can guide learners to pair negative emotional emojis with contextual cues to strengthen the detection of semantic reversal, leveraging their pragmatic transfer advantage from Chinese. For positive irony, it is important to emphasize the importance of emoji‐context congruence, using examples to illustrate that positive emojis can mitigate ambiguity aversion caused by uncertainty avoidance, helping learners accurately grasp the praise intent. Meanwhile, teachers can combine emojis with cultural dimension training, explaining how collectivism enhances sensitivity to contextual cues and how uncertainty avoidance increases comprehension difficulty, to guide learners to consciously use emojis to compensate for cultural differences in expression. Additionally, designing situational simulation activities (e.g., asking learners to select appropriate emojis for given ironic scenarios) can activate their embodied emotional experiences, compensating for the lack of real‐world interaction opportunities in classroom settings.

Several limitations should be noted. First, the sample size (*N* = 28 per experiment) was adequate for the planned repeated‐measures comparisons based on the a priori power analysis, but it nevertheless limits the generalisability of the findings to broader populations of Chinese EFL learners. Replication with larger and more diverse samples (e.g., varying proficiency levels, English‐major students) is needed to confirm the robustness of the observed patterns. Second, the study did not measure emoji familiarity, participants' prior experience with irony, pragmatic competence, or individual differences such as emotional intelligence. These unmeasured variables may moderate the observed effects; future work should include them as covariates or moderators to clarify their contribution. Third, the self‐paced reading paradigm with binary semantic judgments, while standard in L2 irony research (Ellis et al. 2021), provides only endpoint measures of accuracy and overall response speed. It cannot reveal the moment‐by‐moment integration of textual, contextual, and paralinguistic cues, nor can it distinguish between initial literal processing and subsequent inferential processes. Future studies should employ process‐sensitive methods such as eye‐tracking, EEG, or mouse‐tracking to examine the time course of cue integration. Fourth, the emoji‐valence manipulation was validated through a separate rating task with a small group of 21 students, but broader validation across different learner populations and age groups would strengthen the reliability of the materials. Finally, the materials were adapted from Ellis et al. (2021) without a formal norming study for irony detection in Chinese EFL learners, which may affect the ecological validity of the scenarios.

In summary, the current findings for Chinese EFL learners demonstrate that the role of emojis in L2 irony processing is shaped by a unique combination of learning context (classroom‐based instruction and CMC dependence), cultural values (collectivism and uncertainty avoidance), and pragmatic habits (preference for negative irony). This specificity not only enriches the empirical basis of L2 pragmatic research but also provides actionable guidance for improving the effectiveness of English teaching in Chinese contexts.

## 5 Conclusions

This study, framed within the theory of embodied cognition, investigates the impact of emojis on Chinese English learners' comprehension of irony. The findings reveal that emojis can enhance the accuracy of Chinese English learners' comprehension of negative irony and the speed of their semantic judgment of irony. Moreover, the congruity between the emotional valence of emojis and contextual information significantly influences the accuracy of their comprehension of positive irony and their processing speed of irony. These findings suggest that emojis may make speakers' emotional stance more salient and thereby support learners' interpretation of ironic intent, especially when the emotional cue is consistent with contextual information. However, the facilitation of emojis on L2 irony processing is not absolute; instead, it depends on the clarity of contextual information and the congruity between emojis and context. This provides new insights into how emojis influence L2 discourse processing and helps advance the development of theories of L2 discourse processing from the perspective of embodied cognition. Nonetheless, this study has certain limitations. First, it only examines the impact of emojis on irony comprehension in computer‐mediated communication modes, without considering the interaction between learners' individual differences (e.g., emotional intelligence) and emojis in irony processing. Additionally, it fails to account for the influence of nonverbal cues in other modalities (e.g., audiobooks, short videos) on learners' irony processing. Future research can further explore these issues, investigate the impact of nonverbal cues such as emojis on L2 irony processing, and clarify the facilitative role of nonverbal cues in discourse processing within the framework of embodied cognition.

## Funding

The work was funded by the Universiti Teknologi Malaysia Nexus Translational Research (Q.J130000.5553.10G36).

## Ethics Statement

This study was conducted in accordance with the ethical standards of the Declaration of Helsinki and the academic ethics guidelines for human subjects research. The experimental protocol and methodology were formally approved by the Research Ethics Committee of Language Academy, Universiti Teknologi Malaysia (Ethics approval number: No. 2024‐07‐01). All participants were fully informed of the study’s purpose, procedures, potential risks, and rights prior to participation, and all provided written informed consent voluntarily. Participants’ privacy and anonymity were strictly protected: all personal data were de‐identified and securely stored, and no identifiable information was disclosed in the manuscript. Participants had the right to withdraw from the study at any time without negative consequences, and the study involved no procedures causing physical or psychological harm.

## Conflicts of Interest

The authors declare no conflicts of interest.

## Data Availability

The scripts for generating stimuli and performing data analyses of this article are available from the corresponding author via email. The materials and datafiles of this article will be publicly shared upon publication. The complete dataset will be available via the Open Science Framework (https://osf.io/za2qd). None of the experiments were preregistered.
